# Genotype–phenotype correlations and disease mechanisms in *PEX13*-related Zellweger spectrum disorders

**DOI:** 10.1186/s13023-022-02415-5

**Published:** 2022-07-19

**Authors:** Paola Borgia, Simona Baldassari, Nicoletta Pedemonte, Ebba Alkhunaizi, Gianluca D’Onofrio, Domenico Tortora, Elisa Calì, Paolo Scudieri, Ganna Balagura, Ilaria Musante, Maria Cristina Diana, Marina Pedemonte, Maria Stella Vari, Michele Iacomino, Antonella Riva, Roberto Chimenz, Giuseppe D. Mangano, Mohammad Hasan Mohammadi, Mehran Beiraghi Toosi, Farah Ashrafzadeh, Shima Imannezhad, Ehsan Ghayoor Karimiani, Andrea Accogli, Maria Cristina Schiaffino, Mohamad Maghnie, Miguel Angel Soler, Karl Echiverri, Charles K. Abrams, Pasquale Striano, Sara Fortuna, Reza Maroofian, Henry Houlden, Federico Zara, Chiara Fiorillo, Vincenzo Salpietro

**Affiliations:** 1 Department of Neurosciences, Rehabilitation, Ophthalmology, Genetics, Maternal and Child Health, University of Genoa, 16132 Genoa, Italy; 2 Pediatric Neurology and Muscular Diseases Unit, IRCCS Giannina Gaslini Institute, 16147 Genoa, Italy; 3 Unit of Medical Genetics, IRCCS Istituto Giannina Gaslini, 16147 Genoa, Italy; 4 Department of Genetics, North York General Hospital, University of Toronto, Toronto, ON Canada; 5 Neuroradiology Unit, IRCCS Istituto Giannina Gaslini, 16147 Genoa, Italy; 6 Department of Neuromuscular Disorders, Queen Square Institute of Neurology, University College London, London, WC1N 3BG UK; 7 Unit of Pediatric Nephrology and Dialysis, Department of Human Pathology in Adult and Developmental Age “Gaetano Barresi”, University of Messina, Via Consolare Valeria 1, 98125 Messina, Italy; 8 Department Pro.M.I.S.E. “G. D’Alessandro”, University of Palermo, Palermo, Italy; 9 Department of Pediatrics, Zabol University of Medical Sciences, Zabol, Iran; 10 Pediatric Neurology Department, Ghaem Hospital, Mashhad University of Medical Sciences, Mashhad, Iran; 11 Department of Pediatrics, Faculty of Medicine, Mashhad University of Medical Sciences, Mashhad, Iran; 12 Molecular and Clinical Sciences Institute, St. George’s, University of London, Cranmer Terrace, London, SW170RE UK; 13 Innovative Medical Research Center, Mashhad Branch, Islamic Azad University, Mashhad, Iran; 14 Division of Medical Genetics, Department of Specialized Medicine, Montreal Children’s Hospital, McGill University Health Centre (MUHC), Montreal, QC H4A 3J1 Canada; 15 Department of Human Genetics, McGill University, Montreal, QC Canada; 16 Pediatric Clinic and Endocrinology Unit, Department of General and Specialist Pediatric Sciences, University of Genoa, 16147 Genoa, Italy; 17 Computational Modelling of Nanoscale and Biophysical Systems Laboratory, Italian Institute of Technology, 16163 Genoa, Italy; 18 Departments of Neurology and Ophthalmology, University of Kentucky, Lexington, 40506 USA; 19 Department of Neurology and Rehabilitation, College of Medicine, University of Illinois at Chicago, Chicago, IL 60607 USA; 20 Department of Chemical and Pharmaceutical Sciences, University of Trieste, 34134 Trieste, Italy; 21 Department of Biotechnological and Applied Clinical Sciences, University of L’Aquila, 67100 L’Aquila, Italy

**Keywords:** Peroxisome biogenesis disorders, Zellweger spectrum disorder, PEX13; mitochondrial dysfunction

## Abstract

**Background:**

Pathogenic variants in *PEX*-genes can affect peroxisome assembly and function and cause Zellweger spectrum disorders (ZSDs), characterized by variable phenotypes in terms of disease severity, age of onset and clinical presentations. So far, defects in at least 15 *PEX*-genes have been implicated in Mendelian diseases, but in some of the ultra-rare ZSD subtypes genotype–phenotype correlations and disease mechanisms remain elusive.

**Methods:**

We report five families carrying biallelic variants in *PEX13.* The identified variants were initially evaluated by using a combination of computational approaches. Immunofluorescence and complementation studies on patient-derived fibroblasts were performed in two patients to investigate the cellular impact of the identified mutations.

**Results:**

Three out of five families carried a recurrent p.Arg294Trp non-synonymous variant. Individuals affected with *PEX13*-related ZSD presented heterogeneous clinical features, including hypotonia, developmental regression, hearing/vision impairment, progressive spasticity and brain leukodystrophy. Computational predictions highlighted the involvement of the Arg294 residue in PEX13 homodimerization, and the analysis of blind docking predicted that the p.Arg294Trp variant alters the formation of dimers, impairing the stability of the PEX13/PEX14 translocation module. Studies on muscle tissues and patient-derived fibroblasts revealed biochemical alterations of mitochondrial function and identified mislocalized mitochondria and a reduced number of peroxisomes with abnormal PEX13 concentration.

**Conclusions:**

This study expands the phenotypic and mutational spectrum of *PEX13*-related ZSDs and also highlight a variety of disease mechanisms contributing to *PEX13*-related clinical phenotypes, including the emerging contribution of secondary mitochondrial dysfunction to the pathophysiology of ZSDs.

**Supplementary Information:**

The online version contains supplementary material available at 10.1186/s13023-022-02415-5.

## Introduction

Zellweger spectrum disorders (ZSDs) are autosomal recessive conditions caused by genetic defects of PEX proteins (or peroxins)*,* which play crucial roles in several cellular and sub-cellular processes, including peroxisomal membrane formation, the import of peroxisomal matrix proteins and the fission or degradation of peroxisomes [1–3]. Genetic defects in at least 13 of these peroxins (PEX1-3, PEX5-6, PEX10-11B, PEX12-14, PEX16, PEX19, PEX26) may variably affect several of the critical (above mentioned) intracellular steps, leading to a continuum spectrum of clinical phenotypes characterized by variable age of onset and clinical manifestations [2]. These include (i) severe neonatal-onset devastating disorders with profound neurological impairment and frequent multisystemic (e.g., renal, hepatic, skeletal) involvement; (ii) infantile- and pediatric-onset degenerative conditions characterized by slowly progressive neurological deterioration, frequent adrenal dysfunction and abnormal vision and/or sensorineural hearing loss (SHL); (iii) adolescence- or adult-onset isolated visual impairment and/or SHL [3]. *PEX1* and *PEX6* are the most commonly mutated genes in ZSDs, with a frequency of 60.5 and 14.5%, respectively. Conversely, only a few mutations have been identified so far in *PEX13* and limited knowledge on genotype–phenotype correlations and pathogenic mechanism are available for this ultra-rare ZSD subtype [3–7]. The human *PEX13* gene encodes a peroxin part of a docking/translocation module (DTM), that imports peroxisomal matrix proteins during the biogenesis of the organelles [8]. First, PEX13 interacts with itself to form dimers of proteins, a process called homo-oligomerization and then interacts with the PEX14 protein to complete the assembly of the DTM complex at the peroxisomal membrane [9].

Although disease mechanisms implicating PEX13/PEX14 deficiencies in ZSDs are not completely understood, downstream functional impairments of peroxisomes may affect several intracellular pathways, such as very long-chain fatty acids (VLCFA) metabolism, phytanic and pipecolic acid oxidation, or bile acid biosynthesis [10]. Furthermore, an intricated interplay between peroxisomes and mitochondria is also emerging, as both these organelles are actively involved in convergent metabolic processes, such as the metabolism of reactive oxygen species [10–14]. Abnormal mitochondrial function has been identified in muscle biopsies from *PEX12*- and *PEX16*- mutated individuals [15]; in addition, a wide array of mitochondrial abnormalities were identified in different ZSD animal models [13, 16]. These findings from cellular and animal studies highlight a potential, and not yet fully understood, contributory role of mitochondrial dysfunction to pathophysiology of peroxisome biogenesis disorders (PBDs) and ZSD clinical phenotypes.

Herein, we describe six patients from five unrelated families from Italy, Iran, United States and Canada, all affected with ZSDs and found to carry biallelic variants in the *PEX13* gene. A novel p.Arg294Trp non-synonymous variant involving a highly conserved residue within the PEX13 Src homology 3 (SH3) domain was identified in three out of the five families, either in the homozygous or compound heterozygous state (combined with a truncating variant on the other allele). Affected individuals presented a combination of clinical features, including psychomotor delay and/or regression, hypotonia and muscle weakness, visual impairment, SHL and progressive leukodystrophy on brain imaging, with a spectrum of neurological features with variable age of onset and clinical severity. Computational studies based on docking analysis predicted abnormal PEX13 dimerization with impaired DTM complex stability. Cellular studies using patient-derived muscle tissues and fibroblasts revealed both peroxisomal and mitochondrial alterations.

## Materials and methods

### Patients recruitment, clinical and imaging phenotyping

We recruited through international matchmaker platforms, six ZSD patients carrying biallelic *PEX13* variants both in the homozygosity and compound heterozygosity. We compared their clinical, neuroimaging and genetic data with the other *PEX13*-mutated families reported so far (Table 1). Pediatric neurologists and neuroradiologists at different centers reviewed the information from the patients. The families involved in the study provided informed consent for being part of this study. The study was approved by the Ethical Committee of the Gaslini Children’s Hospital and the participating centers.

**Table 1:** Summary of characteristics in *PEX13* variant carriers of this study compared to those reported in literature

Main features	Individual A.II-3	Individual B.II-1	Individual C.II-3	Individual C.II-2	Individual D.II-3	Individual E.II-1	Shimozawa [5]	Shimozawa [27]	Krause [6]	Al-Dirbashi [7]	Al-Dirbashi [7]
Gender	M	F	M	F	M	F	M	NA	F	M	M
Ethnicity	Italian	Canadian	Iraqi	Iraqi	Iranian	Iranian	Caucasian	NA	Turkish	Saudi	Saudi
Diagnosis	ZSD	ZSD	ZSD	ZSD	ZSD	ZSD	ZSD	mild NALD	ZSD	ZSD	ZSD
***PEX13* variant**											
Allele 1	p.Arg294Trp	p.Arg294Trp	p.Arg294Trp	p.Arg294Trp	p.Trp313Ter	p.Gly324Arg	p.Trp234Ter	p. Ile326Thr	p.Trp313Gly	147-KB del	p.G36DfsTer.61
Allele 2	p.Y192QfsTer.14	p.Arg294Trp	partial deletion	partial deletion	p.Trp313Ter	p.Gly324Arg	p.Trp234Ter	p. Ile326Thr	p.Trp313Gly	147-KB del	p.G36DfsTer.61
Psychomotor delay	−	+	−	−	+	+	+	NA	+	+	+
Developmental regression	+	+	+	+	NA	NA	+	NA	NA	NA	NA
Motor impairment	+	+	+	+	+	+	+	NA	+	+	+
Intellectual disability	+	+	−	−	+	+	+	NA	−	+	+
Language impairment	+	+	+	+	+	+	+	NA	−	NA	+
Hearing loss	+	−	-	+	−	+	+	NA	−	−	−
Visual deficit	+	−	−	−	−	+	+	NA	+	−	−
Feeding difficulties	−	+	−	−	−	+	+	NA	−	+	+
Hepatic dysfunction	−	−	−	−	−	−	−	NA	−	−	+
Renal cysts	−	−	−	−	−	−	−	NA	−	−	+
Scoliosis	−	−	+	−	−	−	−	NA	−	−	−
Seizures	−	−	−	−	+	−	−	NA	−	+	+
Deceased	−	−	−	−	+ (20 m)	+ (3 y)	−	NA	+ (31 m)	+ (6 w)	NA
VLCFAs alterations	−	−	−	−	NA	NA	NA	NA	NA	+	+
Muscle biopsy alteration	+	NA	NA	NA	NA	NA	NA	NA	NA	NA	NA
*Brain MRI*											
Cortical malformation	−	−	−	−	+	−	NA	NA	NA	+	NA
White matter hyperintensity	+	+	+	+	−	+	NA	NA	NA	+	NA
Thinning of the corpus callosum	+	NA	+	+	−	NA	NA	NA	NA	NA	NA

*M* male, *F* female, *ZSD* Zellweger spectrum disorder, *NALD* neonatal adrenoleukodystrophy, *NA* not available/not applicable, + present,− not present, *m* months, *y* years, *w* weeks, *VLCFAs* very long chain fatty acids*, MRI* magnetic resonance imaging.

### Genetic studies

Whole-exome sequencing (WES) was performed in the probands and their unaffected parents and the available healthy siblings. Genomic DNA was isolated from 1 ml of peripheral blood using QIAamp DNA Blood Midi kit (Qiagen). Genomic DNA was enriched with SureSelect Clinical research exome 54 Mb (Agilent Technologies). Whole exome sequencing (WES) runs were performed in all affected families on Illumina sequencers, using a standard Illumina pipeline as previously described [17]. Paired-end reads were mapped to the reference human genome sequence (GRch37/hg19), after removal of duplicates. Single-nucleotide polymorphisms (SNPs) and short deletion or insertion (indels) variants were called using the specific variant calling plugin and dbSNP147. The variants were filtered for in-house variants controls and GnomAD databases. Following the pedigree and phenotype, our filtering strategy prioritized rare (< 1% in public databases, including 1000 Genomes project and gnomAD) biallelic and X-linked hemizygous coding variants and/or variants located in genes previously implicated in neurometabolic and/or neurodegenerative disorders. To investigate the presence of homozygosity regions in consanguineous families recruited in this study, homozygosity mapping was performed analyzing the proband variant call format (VCF) WES data on HomozygosityMapper. Validation and segregation studies of the candidate variants that emerged by WES were performed by traditional Sanger sequencing.

### Computational studies

The identified variants were initially evaluated by using a combination of computation approaches. *PEX13 modelling.* Template 1wxu.1.A (Solution structure of the SH3 domain of mouse peroxisomal biogenesis factor 13) [18] with Seq Identity 94.94% (93.67% for the mutant) and coverage from ASN 266 to GLU 344 was used as template for modelling both the wild type (UniProt Q92968) and the mutants domain with Swiss Model [19]. All models underwent the Molecular dynamics protocol below.

*Molecular dynamics* Each model was placed in a cubic box with a water layer of 1.0 nm, neutralised with Na + and/or Cl- ions, and minimised. The steepest descent minimization stopped either when the maximum force was lower than 1000.0 kJ/mol/nm or when 50,000 minimisation steps were performed with 0.005 kJ/mol energy step size, Verlet cutoff scheme, short-range electrostatic cut-off and Van der Waals cut-off of 1.0 nm. AMBER99SB-ILDN force field [20], tip3p water, and periodic boundary conditions were employed. NVT and NPT equilibrations were performed for 100 ps by restraining the protein backbone, followed by 500 ns long NPT production runs at 330 K (250 ns for the complexes). The iteration time step was set to 2 fs with the Verlet integrator and LINCS [21] constraint. End simulation configurations were employed for subsequent docking. All the simulations and their analysis were run as implemented in the Gromacs package v. 2020.3 [22]. Radius of gyration, RMSD, and RMSF have been calculated from configurations sampled every 0.5 ns. Simulations were run on M100 (CINECA, Italy).

*PEX13:PEX14:PEX5 and PEX13:PE13 modelling* PEX14 putative binding site was identified by superposing the Human PEX13 model constructed as described above to the yeast PEX13 (from 1N5Z, also containing the PEX14 peptide [23]) by aligning their alpha carbon with Swiss-PdbWiever 4.1.0. The residues interacting with PEX14 peptide were then identified with LigPlot + (namely: Tyr281, Phe283,Glu290, Val310, GLy312, Trp313, Leu325, Pro327, Asn329, Tyr330). A group of amino acids containing the above residues (from 280 to 330) was then selected as docking sites on PEX13. The human PEX14 (PDB ID 2W84) was then docked to the human PEX13. Being the structure of the human PEX14 in complex with PEX5 known [24] (PDB ID 2W84), this was overlapped to the docking result to obtain the overall complex. We then repeated the process by docking the whole PEX14:PEX5 complex. Finally, a blind docking was performed for the PEX13:PEX14:PEX5 as well as for the PEX13:PEX13 homodimer. The process was repeated for both mutants. Selected models underwent the same molecular dynamics protocol employed above for the PEX13 variants. Docking was performed with HADDOCK 2.4 [25] SASA and distances were calculated as implemented in the Gromacs package v. 2020.3[22].

*Molecular dynamics* Each model was placed in a cubic box with a water layer of 1.0 nm, neutralised with Na+ and/or Cl− ions, and minimised. The steepest descent minimization stopped either when the maximum force was lower than 1000.0 kJ/mol/nm or when 50,000 minimisation steps were performed with 0.005 kJ/mol energy step size, Verlet cutoff scheme, short-range electrostatic cut-off and Van der Waals cut-off of 1.0 nm. AMBER99SB-ILDN force field [20], tip3p water, and periodic boundary conditions were employed. NVT and NPT equilibrations were performed for 100 ps by restraining the protein backbone, followed by 500 ns long NPT production runs at 330 K (250 ns for the complexes). The iteration time step was set to 2 fs with the Verlet integrator and LINCS [21] constraint. All the simulations and their analysis were run as implemented in the Gromacs package v. 2020.3 [22]. Radius of gyration, RMSD, and RMSF have been calculated from configurations sampled every 0.5 ns. Simulations were run on M100 (CINECA, Italy).

### Histopathological and biochemical studies on muscle tissues

One family (Individual A.II-3) provided consent for muscle biopsy, that was performed at the age of 6. Skeletal muscle tissue was obtained from quadriceps muscle, snap-frozen in isopentane and stored in liquid nitrogen. Histological and histochemical methods were performed according to standard procedures, including hematoxylin and eosin, Gomori's trichrome, myofibrillar ATPase (at pH 9.4, 4.6, and 4.3), cytochrome c oxidase (COX), succinate dehydrogenase (SDH), NADH-TR, periodic acid–Schiff (PAS), Oil Red O, acid phosphatase, esterase, and phosphorylase. Immunohistochemical study was performed by indirect immunofluorescence with unspecific findings. The following antibodies were tested: dystrophin COOH, dystrophin Mid Rod, dystrophin NH, alpha-sarcoglycan, beta-sarcoglycan, gamma-sarcoglycan, delta-sarcoglycan, alpha-dystroglycan, merosin, dysferlin, caveolin, and collagen 6. A biochemical assay of the respiratory chain enzymes activity was performed from muscle extracts raccording to Spinazzi et al.[26].

### Immunofluorescence and complementation studies on patient-derived fibroblasts

Skin biopsies were performed upon informed consent in two patients to investigate the cellular impact of the identified candidate mutations. The skin biopsies were performed using the punch procedure and fibroblasts were cultured in RPMI (Gibco) supplemented with 20% (v/v) foetal bovine serum, 2 mM L-glutamine and 1% penicillin/ streptomycin. For control lines, fibroblasts of age-matched normal donors were obtained from the ‘Cell Line and DNA Biobank’ from the Gaslini Children’s Hospital (Genoa, Italy), which is part of the Telethon Network of Genetic Biobanks (project no. GTB12001). All cell lines were tested for mycoplasma contamination (EZ-PCR Mycoplasma Test Kit, Resnova). Cultured fibroblast cell lines were used to carry out immunofluorescence studies and complementation analyses. For all the experiments cells were plated (at a density of 30,000 cells per well) on good-quality clear-bottom 96-well black microplates suitable for high-content imaging. Fibroblast cells were fixed in 10% neutral buffered formalin (05‐01005Q, Bio‐Optica) for 10 min at room temperature (RT). After three washings in phosphate‐buffered saline (PBS), cells were permeabilized with Triton X‐100 0.1% in PBS for 10 min and then blocked with blocking buffer (5% FBS in 0.1% Triton X-100/PBS) for 20 min. Fibroblasts were incubated with primary antibodies at RT for 2 h, after three washes in PBS cells were incubated with secondary antibodies at RT for 1 h. Primary antibodies used for immunofluorescence staining include the following: mouse anti-TOMM20 (Santa Cruz, sc-17764, 1:1000), mouse anti-Pex13 (Santa Cruz, sc-271477, 1:100), rabbit anti- peroxisomal integral membrane protein (PMP70) (Thermo Fisher Scientific, PA1650, 1:1000). Secondary antibodies were conjugated to AlexaFluor 488, AlexaFluor 555, or AlexaFluor 647 (Thermo Fisher Scientific, 1:500). Nuclei were counterstained with 4’, 6-diamodino-2-phenylindole, dihydrochloride (DAPI, Invitrogen by Thermo Fisher Scientific). Negative control samples with only secondary antibodies staining were performed to determine background fluorescence levels. High-content imaging was performed using an Opera Phenix (PerkinElmer) high-content screening system. Wells were imaged in confocal mode, using a 40X water-immersion objective. AlexaFluor 488 signal was laser-excited at 488 nm and the emission wavelengths were collected between 500 and 550 nm. Excitation and emission wavelengths for visualization of AlexaFluor 555 signal were between 570 and 630 nm, respectively. AlexaFluor 647 signal was laser-excited at 640 nm and the emission wavelengths were collected between 650 and 760 nm. Excitation and emission wavelengths for visualization of DAPI signal were 405 and between 435 and 480 nm, respectively. Image analysis of signal intensity, morphology and texture, as well as automatic detection of signal spot, were performed using the Harmony software (version 4.9) of the Opera Phenix high-content system.

### MitoTracker assay (Mitochondria functional assay)

Fibroblast cell lines were grown on a 96-well plate and treated with MitoTraker Red CMXRos (Thermo Fisher Scientific, M7512) 50 nM for 30 min, for dye loading. After treatment, cells were rinsed three times with PBS and fixed in 10% neutral buffered formalin for 10 min, RT and immunofluorescence protocol was performed as above. Cells were stained with mouse anti-TOMM20. DAPI was used for nuclear staining.

### Mitochondrial network distribution analysis

Carbonyl cyanide 4-(trifluoromethoxy) phenylhydrazone (FCCP, Sigma) was used for inhibition of mitochondrial respiration at the concentration of 30 µM for 24 h in the fibroblast culture medium. Dimethyl sulfoxide (DMSO, Sigma) was used as vehicle for untreated condition. After treatment, immunofluorescence protocol was performed as described above. High-content imaging was performed in confocal mode, using a 40X water-immersion objective. Automated image analysis was performed using the Harmony software (version 4.9) of the Opera Phenix, using an automated algorithm, developed using machine-learning techniques. This algorithm allows segmentation of cell cytoplasm into two regions, one comprising the perinuclear area (inner cytoplasmic region) and the second one comprising the more peripheral zone (outer cytoplasmic region). Further image analysis of mitochondrial network distribution, based on TOMM20 and/or Mitotracker signal morphology, intensity, density and texture, was then performed using the Harmony software as described above.

## Results

We report clinical and molecular findings in six individuals affected with a spectrum of neurological features and biallelic pathogenic variants affecting the *PEX13* locus*.*

### Molecular findings

WES revealed three compound heterozygous and three homozygous variants in *PEX13* (Table 1). Variants were defined using the NM_002618 transcript. WES detected a compound heterozygous state for a truncating (NM_002618: c.573_574delTT;p.Y192Ter) variant and a missense (NM_002618: c.880C>T;p.Arg294Trp) variant; a homozygous (NM_002618: c.880C>T;p.Arg294Trp) variant; heterozygosity for a partial deletion in *PEX13* and a missense (NM_002618: c.880C>T;p.Arg294Trp) variant in two siblings; a homozygous truncating (NM_002618: c.938 G>A; p.Trp313Ter) variant, and a homozygous missense (NM_002618: c.970 G>C; p.Gly324Arg) variant. The missense variants (p.Arg294Trp and p.Gly324Arg) were classified as damaging by SIFT, PolyPhen-2 and Mutation Taster, with an average 31 CADD Score. None of the variants identified was present in a homozygous state in the in-house database, GnomAD dataset (https://gnomad.broadinstitute.org), UK Biobank and Queen Square Genomics. Sanger sequencing of the patients and the parents confirmed this result and showed that the parents were carriers for those variants.

### Demographic data

**Fig. 1:**
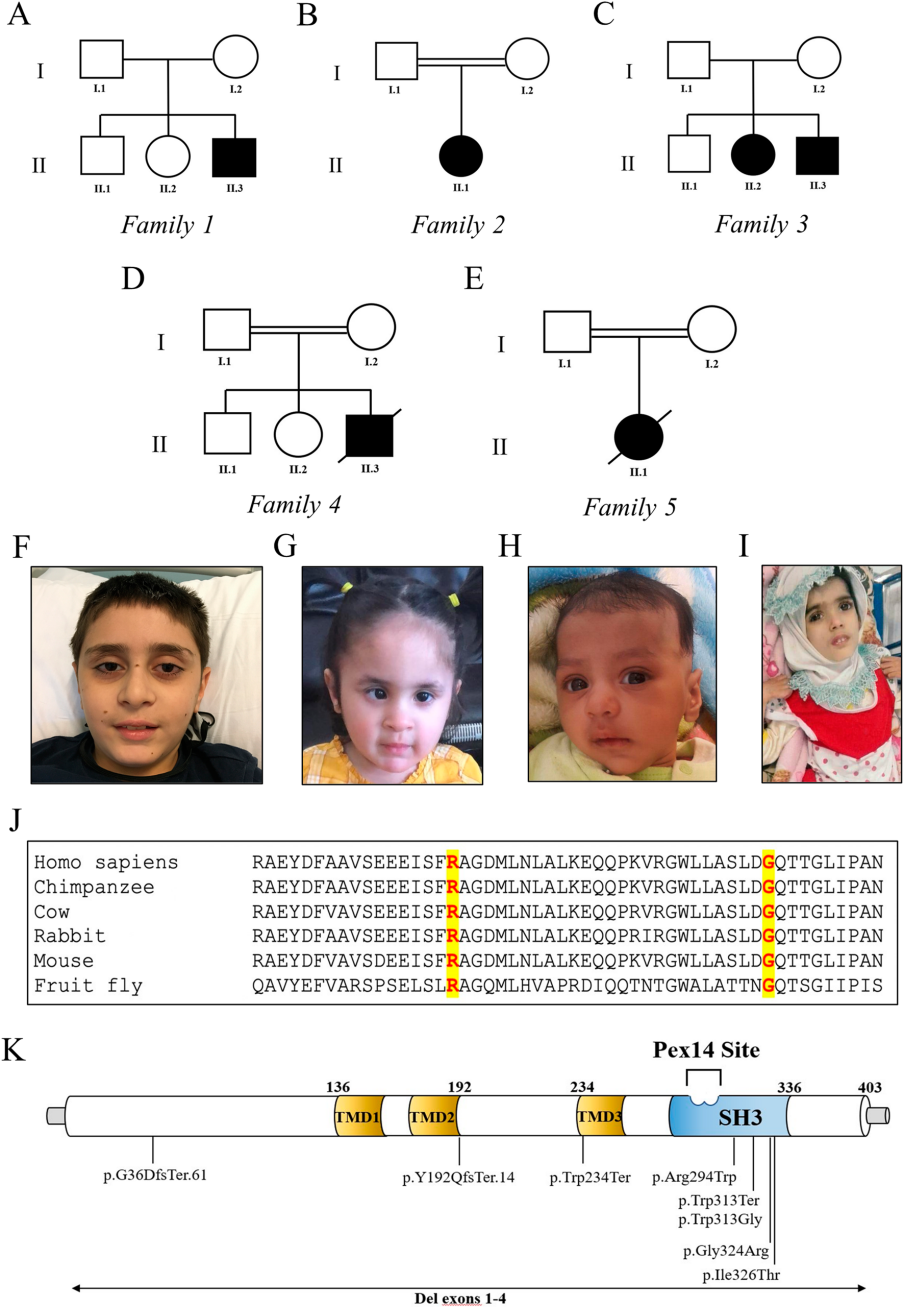
Family trees, *PEX13*-associated clinical features, PEX13-interspecies alignment, PEX13 protein and *PEX13*-associated mutations. The pedigree diagrams of six families carrying *PEX13* variants (**A**–**E**). *PEX13*-associated clinical features of individuals A.II-3 (**F**), B.II-1 (**G**), D.II-3 (**H**), and E.II-1 (**I**). Interspecies alignment of PEX13 protein sequences (**J**) generated with Clustal Omega (https://www.ebi.ac.uk) shows that p.Arg294Trp and p.Gly324Arg missense variants identified in this study occur at residues highly conserved across species (highlighted in yellow). Schematic of the human PEX13 protein indicating the positions of the variants identified so far (**K**)

Six affected children from five unrelated families were identified with either compound heterozygous or homozygous changes in *PEX13*. Three out of them were male (50%) and three were females (50%). Among the five families, three were consanguineous (Fig. 1).

### Phenotypes of PEX13-related ZSDs

#### Family 1

**Fig. 2:**
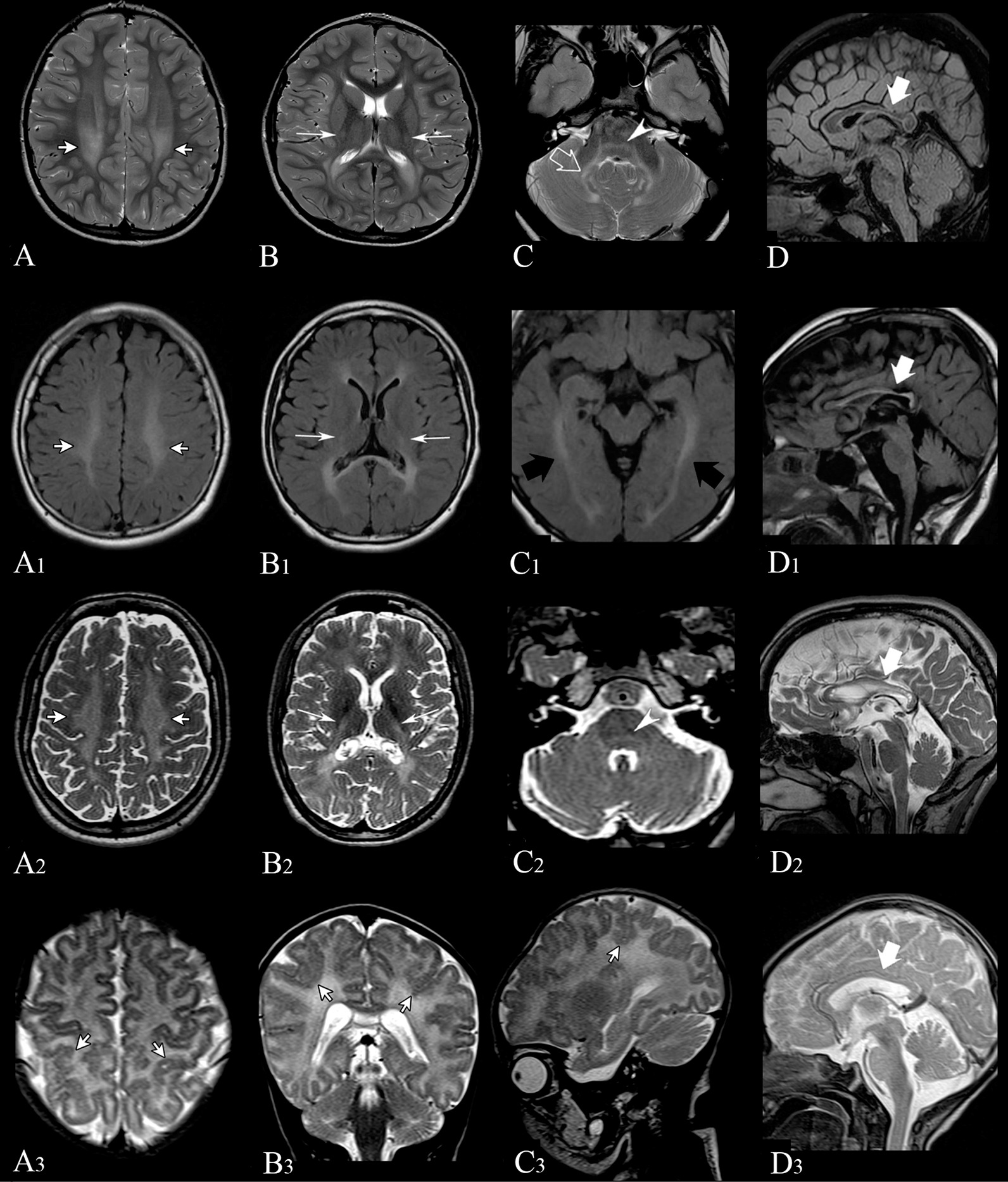
Brain MRI images of individual A.II-3 (**A**–**D**) at 8 years of age. Axial (**A**–**C**) T2-weighted images show bilateral hyperintensity within the posterior periventricular white matter (short white arrows in A), posterior limb of the internal capsules (long white arrows in **B**), within the cerebellar peduncles and dentate nuclei in the cerebellar region (empty white arrow in **C**) and in medial lemnisci (white arrowhead in **C**). Sagittal FLAIR image (**D**) shows thinning of the posterior portions of the corpus callosum (white arrow). Brain MRI images of individual C.II-3 (**A1**–**D1**) at 11 years of age. Axial (**A1**–**C1**) FLAIR images show bilateral hyperintensity within the posterior periventricular white matter (short white arrows in **A1**), posterior limb of the internal capsules (long white arrows in **B1**), and optic radiations (black arrows in **C1**). Sagittal FLAIR image (**D1**) shows thinning of the corpus callosum (white arrow). Brain MRI images of individual C.II-2 (**A2**–**A2**) at 16 years of age. Axial (**A2**–**C2**) T2-weighted images show bilateral hyperintensity within the posterior periventricular white matter (short white arrows in **A2**), posterior limb of the internal capsules (long white arrows in **B2**), and in medial lemnisci (white arrowhead in **C2**). Sagittal T2-weighted image (**D2**) shows thinning of the corpus callosum (white arrow). Brain MRI images of Individual D.II-3 (**A3**–**D3**) at 1 month of age. Axial (**A3**), Coronal (**B3**), and Sagittal (**C3**) T2-weighted images show bilateral malformation of cortical development in parietal lobes, with a polymicrogyria-like appearance (short white arrows). Midline Sagittal T2-weighted image (**D3**) does not show thinning of the corpus callosum at this early stage of life (white arrow)

**Fig. 3:**
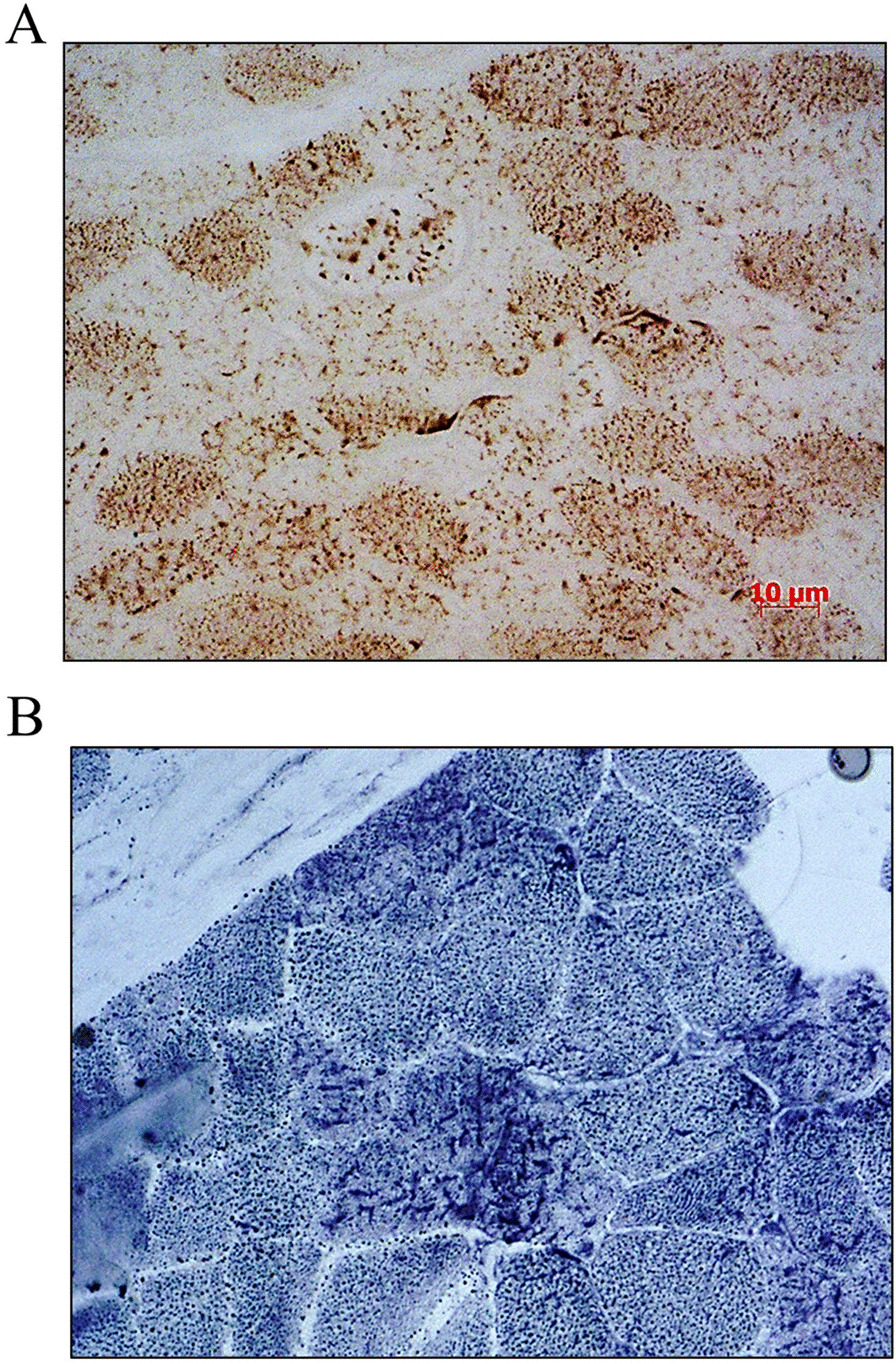
Muscle biopsy from Individual A-II.3. COX (**A**) and SDH (**B**) histochemical stain showed an uneven distribution of mitochondria including patchy or reticular patterns and areas devoid of oxidative staining. At higher magnification histopathological observation mitochondria appears also larger and possibly swollen (3A-B)

Individual A.II-3 is the third child of healthy, non-consanguineous Italian parents (Fig. 1). Family history was unremarkable. He was delivered by Cesarean with a birth weight of 3400 g (50th percentile) at 40 weeks gestation (GW). He did not display distinctive craniofacial features (Fig. 1). The boy pronounced his first two-word sentences at age 36 months, in the context of normal psychomotor development. In early childhood, he required hearing aids bilaterally due to sensorineural hearing impairment and he suffered from a progressive decrease in visual acuity, due to severe myopia. At the age of 5, motor achievements were progressively lost until he became wheelchair-bound at age of 7 due to spasticity and ataxia. His neurological examination revealed diffuse muscular hypotrophy, spastic tetraparesis predominantly affecting the lower limbs, dystonic posture of lower extremities, increased deep tendon reflexes, sustained bilateral ankle clonus, spontaneous Babinski sign, and cerebellar features including dysmetria, dysarthria, nystagmus and intention tremors (Additional file 2: Video 1). He showed mild cognitive regression, without other signs of extra-neurological involvement. Brain Magnetic Resonance Imaging (MRI) at the age of 6 showed bilateral hyperintensity within the posterior periventricular white matter and the dentate nuclei. Follow-up MRI at 8 years of age, revealed extending of white matter signal abnormalities to the brainstem (Fig. 2). Plasma VLCFA analysis documented only minimally altered values of C26:0 and C24:0/C22:0 ratio (see Additional file 1: Table S2). Muscle biopsy showed an irregular and punctate mitochondrial network at COX and SDH staining with subsarcolemmal aggregates, consistent with mitochondrial alterations (Fig. 3). Muscle respiratory chain enzyme (RCE) activities indicated a significantly decreased cytochrome C oxidase activity [1.59 (normal values 1.80–2.45)]. Genetic analysis identified a compound heterozygous mutation in *PEX13*, a truncating (NM_002618: c.573_574delTT;p.Y192Ter) variant inherited from the mother and a missense variant (NM_002618: c.880C>T;p.Arg294Trp), inherited from the father (Additional file 1: Fig. S1). At the last follow-up at 9 years of age, individual A.II-3 is wheelchair-bound but able to take a few steps with support (Additional file 2: Video 2) and to communicate with short sentences. He is currently receiving baclofen (25 mg twice/day).

#### Family 2

Individual B.II-1 is a 4-years old female child of healthy first-cousin Pakistani-Canadian parents (Fig. 1). She was born vaginally at 40 GW with a birth weight of 1500 g (< 3rd percentile). At birth, she had profound hypotonia, feeding difficulties and failure to thrive. At age 3 years of age, her growth parameters showed severe growth retardation with a weight of 8.9 kg (< 3rd percentile) and height of 78 cm (< 3rd percentile). Craniofacial features showed high forehead and strabismus (Fig. 1). Further developmental skills showed global developmental delay. She was able to cruise but not able to walk. She was non-verbal with poor receptive language and poor fine motor skills. Brain MRI revealed diffuse hypomyelination with abnormal confluent FLAIR hyperintense signal abnormality, particularly involving the cerebellar white matter. Extensive cerebellar atrophy and pontine/vermian hypoplasia were also reported. VLCFA assay on patient-derived fibroblasts showed marginal elevation of C26:0 with a normal C26:0/C22:0 ratio; catalase immunofluorescence microscopy analysis on patient-derived fibroblasts indicated less import-competent peroxisomes at 40 °C, consistent with an underlying peroxisomal biogenesis disorder. WES revealed a homozygous variant of *PEX13* (NM_002618: c.880C>T;p.Arg294Trp). Sanger sequencing of the patients and the parents confirmed this result and showed that the parents were carriers for this variant. The child is currently 4 years of age and only able to tolerate soft diet. There has been no regression, no new gain of developmental skills and still non-verbal.

#### Family 3

Individual C.II-3 is a 19-year-old boy, first evaluated at age of 11 for neurodevelopmental regression since he was 3. He is the third child of non-consanguineous Iraqi parents (Fig. 1), born full-term with a birth weight of 3.5 kg, following an uneventful pregnancy. His development was normal in the first two years of life. At the age of 3, parents noticed an unsteady gait resulting in frequent falls in addition to speech regression by age of 5. Nevertheless, language comprehension and cognitive function remained unaffected. At the age of 7, he became wheelchair dependent. He exhibited spastic quadriparesis with contractures of the wrists, fingers and ankles. He retained the ability to grasp small objects. He suffered from scoliosis, which was surgically corrected at the age of 13. Moreover, physical examination showed cranial nerve abnormalities including right eye exotropia and multidirectional nystagmus. Brain MRI performed at the age of 11 documented bilateral hyperintensity within the posterior periventricular white matter, posterior limb of the internal capsules and optic radiations, and thinning of the corpus callosum (Fig. 2). WES revealed compound heterozygosity for a maternally inherited partial deletion and a paternally inherited missense (NM_002618: c.880C>T;p.Arg294Trp) variant in the *PEX13* gene. Plasma VLCFA analysis, phytanic acid and pristanate were normal (Additional file 1: Table S1).

Individual C.II-2 is the older sister of Individual C.II-3 (Fig. 1). She was born at term with normal birth weight. She was referred at age 23 years old for progressive spastic paraparesis, starting at the age of 12. Neurodevelopmental milestones were achieved at the expected time. Indeed, her parents had no concerns until the age of 10 when the patient began having hearing loss requiring hearing aids. At the age of 11, she exhibited bilateral upper extremity kinetic tremors, and by age 12 she started having difficulty keeping up with her peers. By the age of 16, she required a walker to ambulate and was eventually confined to a wheelchair. Additionally, neurological examination revealed horizontal nystagmus with left gaze, generalized spasticity with muscle atrophy worse distally, reduced sensation to vibration and pinprick from the feet up to the ankles, ankle and contractures of toes. Besides, nerve conduction studies of the right arm and leg showed uniform demyelination. Brain MRI documented bilateral hyperintensity within the posterior periventricular white matter, posterior limb of the internal capsules, and medial lemnisci**,** and thinning of the corpus callosum (Fig. 2). WES detected compound heterozygosity for a maternally inherited partial deletion and a paternally inherited missense (NM_002618: c.880C>T; p.Arg294Trp) variant in the *PEX13* gene. VLCFA and pipecolic acid dosages were both within normal limits (Additional file 1: Table S1). In the hypothesis she might have an underlying mitochondrial disorder, she was trialled on coenzyme Q10 supplementation, without benefit. She is currently taking cholic acid and baclofen three times a day. She was fitted with ankle–foot orthoses and received botulinum toxin injections in the lower extremities, without improving her spasticity. She has retained the ability to use her hands to use cutlery, write, manipulate a phone, and propel her manual wheelchair.

#### Family 4

Individual D.II-3 was the third-born child of healthy consanguineous Iranian parents (Fig. 1). Family history was not significant. He was born at 39 GW via caesarean section, with a birth weight of 3440 g, head circumference of 35 cm and length of 53 cm. APGAR score was 9/10. A few hours after birth, he presented the first episode of seizures, for which phenobarbital (20 mg/Kg/day) was administered. Another episode occurred after 7 days. Blood and urine cultures were negative, C-reactive protein (CRP) was 55 mg/L. Ceftriaxone and phenobarbital were administered for 4 days and phenobarbital was continued at home. Brain MRI, performed at one month of age, showed bilateral malformation of cortical development in parietal lobes, with a polymicrogyria-like appearance (Fig. 2). At last follow-up (age 12 months), he was severely hypotonic with head lag. The patient experienced different seizures types, including myoclonic and tonic seizures. Electroencephalography (EEG) showed multifocal sharp waves (Additional file 1: Fig. S2). Genetic analysis revealed a homozygous variant in *PEX13* (NM_002618: c.938 G>A; p.Trp313Ter). This variant was confirmed by Sanger sequencing and identified in both unaffected parents and the two healthy siblings in the heterozygous state. Individual D.II-3 deceased at 1 year and 8 months of age due to poor feeding and severe breathing difficulties.

#### Family 5

Individual E.II-1 was the only child of a healthy consanguineous Iranian couple (Fig. 1). No antecedents of neurological diseases had ever been mentioned. Extended metabolic screening at birth was negative. After normal psychomotor development, she presented at 1 year of age with regression of neurodevelopmental milestones, loss of eye contact and cognitive impairment. She developed mild axial and limbs dystonia, dysarthria, hearing loss, visual fixation and gaze impairment. Brain MRI showed mildly high T2 and FLAIR signals in the cerebellar peduncles and the cerebellar and cerebral white matter (data not shown). Fundoscopy examination showed a cherry-red spot of the macula and diffuse white dots, while irregularities of the retinal pigment epithelium were present at Optic Coherence Tomography (Additional file 1: Fig. S3). Metabolic studies revealed elevated succinic acid (5840 mmol/mol creatinine). WES identified a homozygous missense variant in *PEX13* (NM_002618: c.970 G>C; p.Gly324Arg). The patient died at 3 years of age due to respiratory difficulties.

### Results from computational simulations

**Fig. 4:**
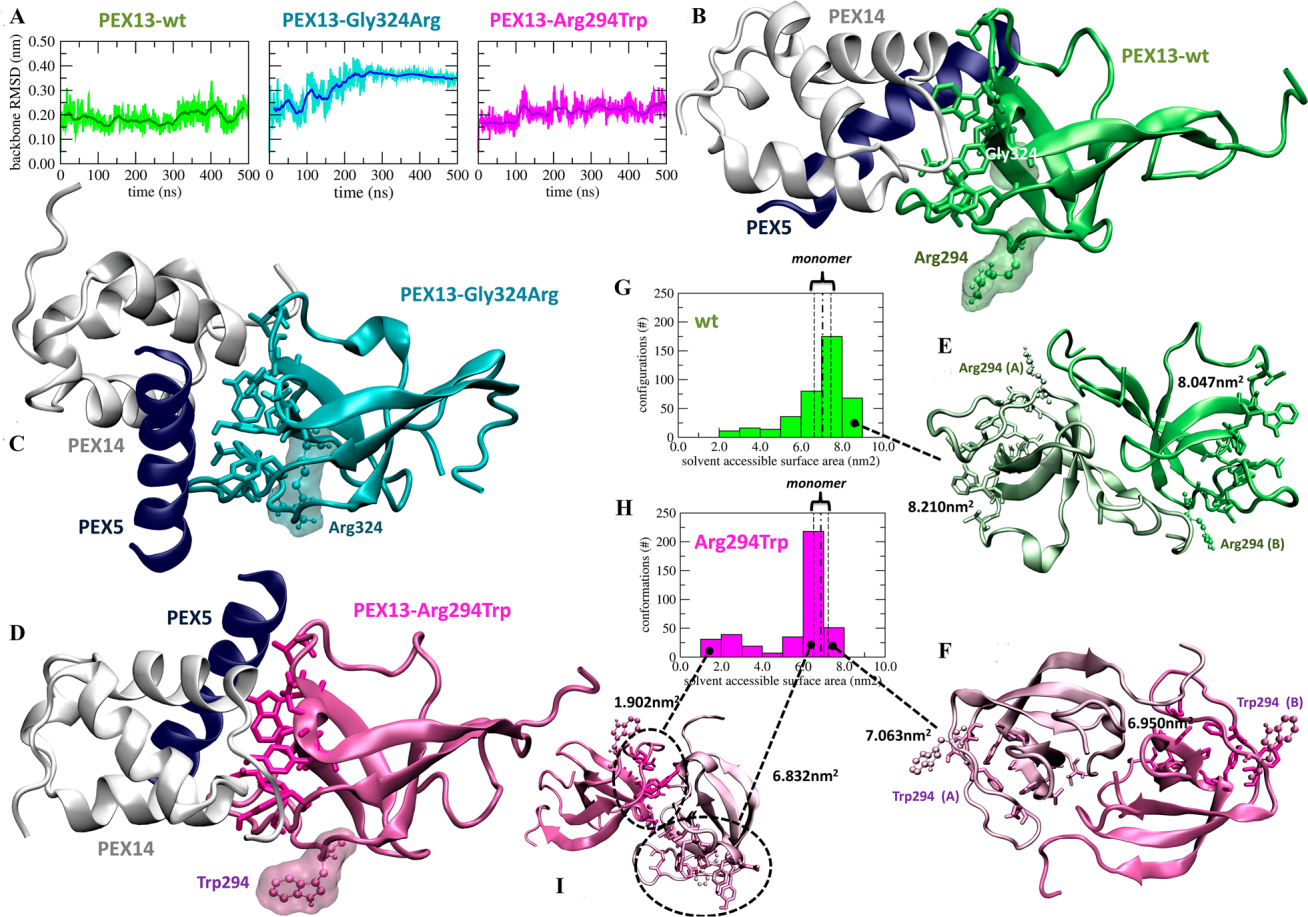
Molecular modelling of identified *PEX13* missense variants (p.Arg294Trp and p.Gly324Arg). **A** Analysis of 500 ns of atomistic molecular dynamics trajectories run in water solvent at 330 K, protein backbone RMSD for the wild type PEX13 (left), the mutant Gly324Arg (center), and its Glu294Trp mutant (right), running averages over 50 data points are highlighted (dark solid lines); **B** PEX13-wt:PEX14:PEX5 tetramer; **C** PEX13-Gly324Arg:PEX14:PEX5 tetramer; **D** PEX13-Arg294Trp:PEX14:PEX5 tetramer; **E** PEX13-wt:PEX13-WT homodimer; **F** PEX13-Arg294Trp:PEX13Arg294Trp homodimer; **G**–**H** solvent accessible surface area distribution of the residues responsible to the binding of PEX13 with PEX14 for PEX13-wt:PEX13-wt (**G**) and PEX13-Arg294Trp:PEX13-Arg294Trp (**H**), the distributions were calculated over both dimers of all configurations generated by docking, the monomers average value (dotted dashed lines) as well as its standard deviation (dashed lines) calculated over configurations sampled along 500 ns of molecular dynamics simulations are also indicated; **I** an aberrant PEX13-Arg294Trp:PEX13-Arg294Trp homodimers in which one of PEX14 binding site is buried due to dimerisation. Configurations were obtained by PEX13 homology modelling followed by 500 ns of molecular dynamics simulations and (**B**–**D**) docking to PEX14:PEX5 (**E**–**I**) blind docking to PEX13. The residues predicted to be involved in PEX13:PEX14 interactions (and selected binding site for the dockings of panels **B**–**D**) are highlighted (licorice), their solvent accessible surface area is labelled in panels E, F, I. Arg294 and Trp294 are highlighted in PEX13-wt and PEX13-Arg294Trp, respectively; Gly324 in PEX13-wt (**B**), and Arg324 in PEX13-Gly324Arg (**C**). Color code: PEX13-wt (green), PEX13-Gly324Arg (cyan), PEX13-Arg294Trp (magenta), PEX14 (white), PEX5 (blue)

PEX13 is known to interact with PEX14 and PEX5 [27]. Computational predictions showed that the folding of PEX13 is affected by the presence of p.Gly324Arg while not by p.Arg294Trp as assessed by comparing their average backbone displacement from the wild type conformation, measured by the root mean squared deviation, along 500 ns long molecular dynamics simulations of each protein (Fig. 4A). Computational results based on protein–protein docking, suggested the misfolded PEX13- p.Gly324Arg to be unable to form the expected complex with PEX14 and PEX5 (Fig. 4B, C). Instead PEX13-p.Arg294Trp was shown capable of keeping the same backbone structure as the wild type (Fig. 4A). Also, the residue 294 does not interact directly with either PEX14 or PEX5 and the observed PEX13:PEX14:PEX5 assembly closely resembles that formed with the wild type (Fig. 4D). Further, residue 294 does not seem involved in the stabilization of an aberrant PEX13:PEX14:PEX5 assembly (Additional file 1: Fig. S4), indicating to look for the root reasons of its malfunctioning elsewhere.

It was suggested that PEX13 could dimerize [1]. In this framework our analysis suggests that the role of PEX13 dimerization is that of exposing the residues responsible for its interaction with PEX14 making its binding site more accessible (Fig. 4E), and enabling their interaction. The statistical analysis of docking results showed that Arg294 is involved in PEX13 dimerization more often than Trp294 (Additional file 1: Figs. 5–6) while the mutation p.Arg294Trp inhibits the process allowing the preferential formation of aberrant dimers that covers PEX14 binding site (Fig. 4F). Indeed the mutation p.Arg294Trp reduces the surface area accessible to PEX14 (Fig. 4G–H), and enables the formation of PEX13:PEX14 homodimers where at least one partner is unable to bind PEX14 (Fig. 4I).

### Muscle studies reveal biochemical alterations of mitochondrial function

Muscle biopsy from Individual A-II.3 showed in several myofibers at COX and SDH staining an uneven distribution of mitochondria including patchy or reticular patterns and areas devoid of oxidative staining. These areas were prevalent in the centre of the fibres, whereas mitochondrial aggregates were evident in the subsarcolemmal regions of the fibre. These findings point toward a defect in the correct distribution of mitochondria within the muscle fibre. At higher magnification histopathological observation mitochondria appears also larger and possibly swollen (Fig. 3A–B).

### Reduced peroxisomes in fibroblasts derived from patients bearing PEX13

**Fig. 5:**
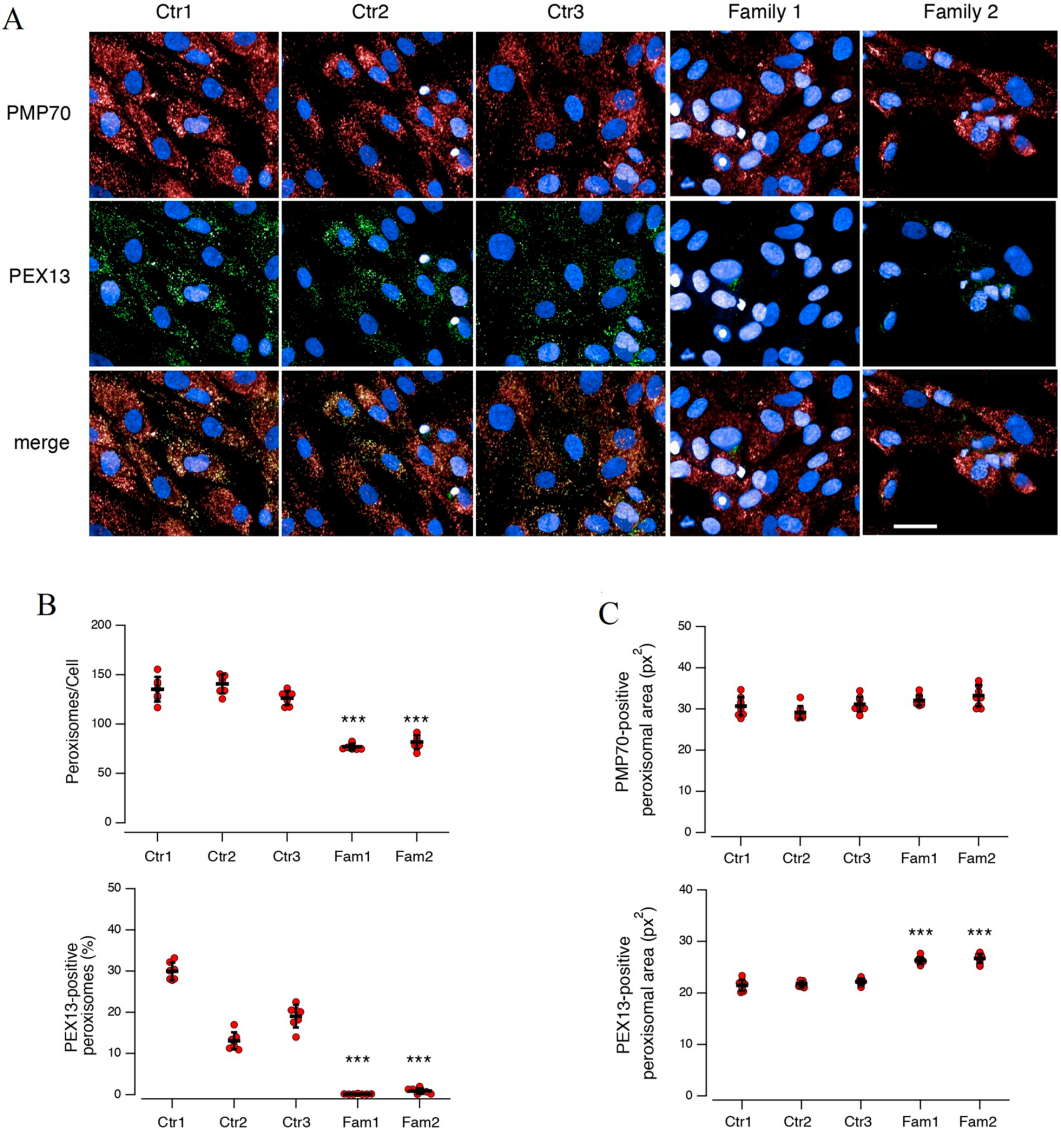
Reduced peroxisomes in fibroblasts derived from Individuals A.II-3 and B.II-1*.* Images showing PMP70- (**A**, upper panels) and PEX13-positive (**A**, middle panels) peroxisomes. PEX13 localizes in a subset of PMP70-positive peroxisomes (**A**, bottom panels). Quantification of PMP70- (**B**, top graph) and PEX13-positive (**B**, bottom graph) peroxisomes in fibroblasts: ZSD patients display fewer PMP70-positive peroxisomes and severely impaired expression of PEX13-positive peroxisomes. Each dot represents the value obtained from the analysis of a different biological replicate, in which 200 cells were imaged and analyzed. Lines indicate means ± SD, n = 50. Morphological analysis of PMP70- (**C**, top graph) and PEX13-positive (**C**, bottom graph) peroxisomes in fibroblasts: ZSD patients display enlarged PEX13-positive peroxisomes, while the size of overall PMP70-positive peroxisomes is not affected. Each dot represents the value obtained from the analysis of a different region (having area of 0.1 mm^2^) in which all PMP70- or PEX13-positive peroxisomes were imaged and analyzed. Lines indicate means ± SD, n = 9. Symbols indicate statistical significance versus pooled values of controls: ***, *p* < 0.001. Scale bar: 50 µm

Fibroblast from Individuals A.II-3 and B-II-1 were examined, and the effect of variants were compared. ZSD patients display fewer PMP70-positive peroxisomes and severely impaired expression of PEX13-positive peroxisomes (Fig. 5). Moreover, ZSD patients exhibit enlarged PEX13-positive peroxisomes, while the size of overall PMP70-positive peroxisomes is not affected (Fig. 5).

### Impaired mitochondrial network and localization in PEX13-mutant fibroblasts

**Fig. 6:**
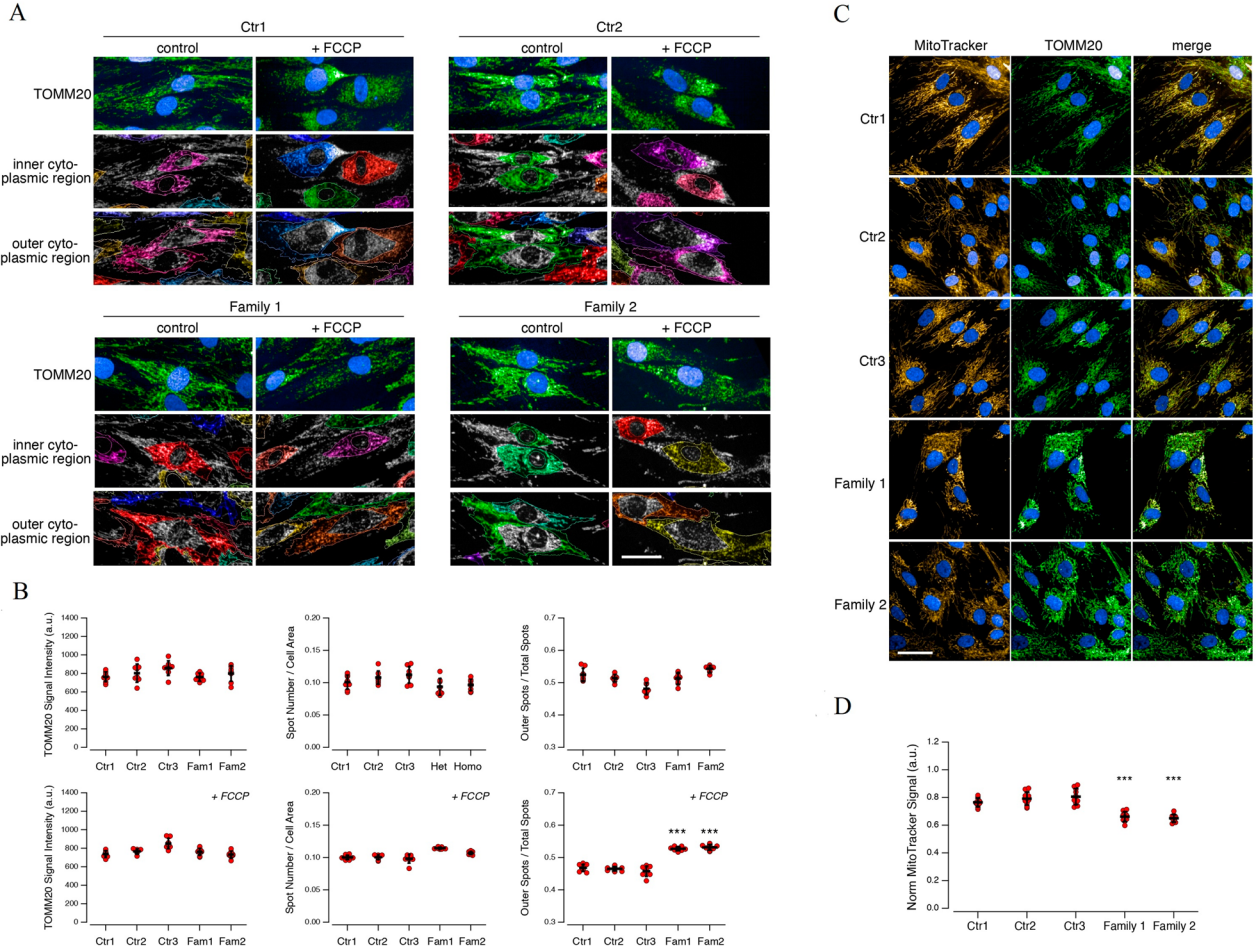
Impaired mitochondrial network and localization in fibroblasts derived from Individuals A.II-3 and B.II-1. Images showing, for each individual, the mitochondrial network in cells under resting condition (DMSO-treated cells; **A**, upper left panels) or treated with the mitochondrial uncoupler FCCP (30 µM; **A**, upper right panels). Mitochondria were visualized by staining for the mitochondrial marker TOMM20. Cytoplasm was divided into inner (**A**, middle panels) and outer (**A**, bottom panels) cytoplasmic regions. Quantification of total TOMM20 signal intensity (**B**, left graphs), number of spots (resembling individual mitochondria) normalized per cell area (**B**, middle graphs), and percentage of spots localized in the outer cytoplasmic region (**B**, right graphs) in cells under resting condition (DMSO-treated cells; top) or treated with FCCP (30 µM; Fig. 6B, bottom). In ZSD cells, the percentage of mitochondria that are mislocalized in the outer cytoplasmic region, following stress conditions, is markedly increased. Each dot represents the value obtained from the analysis of a different biological replicate, in which 500 cells were imaged and analyzed. Lines indicate means ± SD, n = 9. Images (**C**) showing the mitochondrial network visualized by staining for the mitochondrial marker TOMM20 or using the fluorescent dye MitoTracker, which accumulates in viable mitochondria dependently on mitochondria membrane potential. Quantification of total MitoTracker signal (corresponding to the dye accumulated into mitochondria) normalized for TOMM20 signal (**D**). ZSD patients display decreased MitoTracker accumulation. Each dot represents the value obtained from the analysis of a different biological replicate, in which 250 cells were imaged and analyzed. Lines indicate means ± SD, n = 12. Symbols indicate statistical significance versus pooled values of controls: ***, *p* < 0.001. Scale bar: 50 µm

In patient-derived fibroblast cell lines, the percentage of mitochondria that are mislocalized in the outer cytoplasmic region, following stress condition, is markedly increased (Fig. 6). Quantification of total MitoTracker signal (corresponding to the dye accumulated into mitochondria) normalized for TOMM20 signal revealed a decreased MitoTracker accumulation in ZSD patients (Fig. 6).

## Discussion

Variants in the *PEX13* gene are implicated in one among the rarest form of ZSDs. We report six patients affected by Zellweger spectrum disorder, ranging from moderate to severe clinical phenotype, carrying either truncating or missense variants in the *PEX13* gene. A novel missense variant (NM_002618: c.880C>T; p.Arg294Trp) was identified in three out of five families either in the homozygous and the compound heterozygous state*.* Only a few individuals harboring *PEX13* variants have been reported in the literature so far (Table 1). Shimozawa et al. [5] described [1] a patient with early-onset severe generalized paresis, sensory losses and gavage feeding due to a nonsense mutation in homozygosity (NM_002618: c.702G>A; p.Trp234Ter), which resulted in the loss of the transmembrane domain and SH3 domain of the PEX13 protein and [2] a more mildly affected patient with neonatal adrenoleukodystrophy (NALD), homozygous for a missense variant affecting a conserved residue (p.Ile326Thr) within the SH3 domain of PEX13 [4, 5, 28]. Krause et al. reported a Turkish female individual found to carry a homozygous p.Trp313Gly variant also located within the PEX13 SH3 domain [6]. The child presented with hypotonia and progressive motor impairment and died at the age of 31 months of age. The latest report identified two homozygous deletion variants, that included the *PEX13* gene, in Saudi boys affected with severe neonatal-onset hypotonia, seizures, hepatic dysfunction and death within the first months of life [7]. In our study, individuals carrying the missense p*.*Arg294Trp and p.Gly324Arg variants in the compound heterozygous state (and a loss of function variant on the other allele) displayed a milder clinical severity, characterized by psychomotor regression and late-onset leukodystrophy, compared to Individual B.II-1 and Individual E.II-1 that were found homozygous for the p*.*Arg294Trp variant and p.Gly324Arg variant, respectively. Individual D.II-3, carrying the p.Trp313Ter homozygous truncating variant, was affected by a severe neonatal-onset phenotype and suffered from epilepsy (myoclonic and tonic types) since birth. Individual C.II-3 showed skeletal involvement (scoliosis). None of the affected individuals presented other systemic features (*i.e.*, liver dysfunction, adrenal insufficiency and renal oxalate stones).

Neuroradiological features suggestive of ZSDs include white matter signal abnormalities with or without malformations of cortical development (e.g., polymicrogyria, pachygyria), and corpus callosal dysgenesis [29]. Accordingly, available MRI scans of our patients detected diffuse disorder of myelination with peculiar involvement of dentate nuclei and peri-dentate white matter and abnormalities of the corpus callosum; the polymicrogyria was identified only in the individual D.II-3, who carried a truncating variant in homozygosity (Fig. 2).

In all the affected individuals from this cohort who underwent detailed metabolic work-up, VLCFA levels resulted within normal limits. This finding is consistent with previous studies of ZSDs due to genetic defects in different peroxins that were found with normal serum VLCFA [30–33]. Our study further highlights the importance of genetic screening targeting peroxisomal disorders, even though plasma peroxisomal metabolites are unremarkable, in case of moderate clinical presentations of ZSDs (e.g., visual and hearing impairment, progressive psychomotor regression, white matter abnormalities on brain MRI).

Notably, normal VLCFAs possibly indicate that a defect of beta-oxidation does not represent the main pathomechanism underlying ZSDs, thus pointing toward other cellular defects, such as mitochondrial dysfunction. Various mitochondrial abnormalities, such as the presence of mitochondrial inclusions and the accumulation of enlarged bizarre mitochondria, were previously reported in muscle tissues from patients with a presumptive and/or a confirmed diagnosis of ZSD [15, 34–37], in patients with *PEX16* and *PEX12* mutations [15] and in a mouse model carrying a *PEX13* deletion, which led to aberrant accumulation of mitochondria in brain tissue [13]. In this report, functional studies on fibroblasts from ZSD patients carrying the *PEX13* p.Arg294Trp variant (either in the compound heterozygous or the homozygous state) revealed a reduced number of PEX13 expressing, enlarged peroxisomes as well as a mitochondria mislocalization, that was triggered under particular cellular stress condition. Furthermore, our studies on muscle tissues from individual A.II-3 revealed an uneven distribution of mitochondria, and central areas devoid of oxidative staining. These findings suggest secondary mitochondrial dysfunction and altered distribution of mitochondria in the muscles as the result of the peroxisomal deficiency (Fig. 3). The mitochondrial pathology in PBD reported here can be partly explained by the recent molecular experiments in a yeast model and in fibroblasts of *PEX3*-mutant ZSD patient [38]. The authors identified that the peroxins, in condition of lack of peroxisomes in PBD, reach the mitochondrial membrane, retaining the ability of assemble and import peroxisomal proteins into a non-native organelle [38]. This leads to an impaired mitochondrial structure and function.

One of the variants reported here substitutes the Arginine at position 294 for a Tryptophan and is located in the SH3 domain, which represents an important locus to interaction between PEX5 and PEX14 during the biogenesis of the peroxisomes [9, 27]. However, our understanding of the specific domains involved in peroxins interactions is still incomplete. Our computational predictions showed that the presence of p*.*Arg294Trp drives the formation of aberrant dimers incapable of exposing as efficiently the residues responsible for its binding with PEX14. These findings are consistent with previously functional analysis performed on the fibroblasts with *PEX13* p.Trp313Gly variant, also sited within the SH3 domain, which revealed interference with PEX13 homo-oligomerization [1]. Therefore, disease mechanisms in p.Arg294Trp variant may implicate the defective homo-oligomerization of PEX13.

## Conclusions

Despite the current hypothesis that severe ZSD patients tend to carry severe loss-of-function mutations such as nonsense mutations, frameshifts, and deletions [39], our study reveals the most clinically severe phenotype in terms of earliest age of onset and marked neurological impairment occurred in individuals harboring missense p*.*Arg294Trp and p.Gly324Arg variants in homozygosity, and not in those carrying the same missense variants in the compound heterozygous state (associated with a truncating variant). Taken together, our results suggest that *PEX13* variants identified in this study may give rise to impaired peroxins interactions and localization as well as mitochondrial abnormalities, leading to an ineffective peroxisome biosynthetic pathway. Future studies, using both cellular and animal models, should be performed to precisely assess the impact of different peroxin gene mutations on specific biochemical (peroxisomal and mitochondrial) functions, to possibly develop therapeutical strategies targeted at individual disease mechanisms.

## Supplementary Information

**Additional file 1: Additional Table 1.** Plasma biochemical analyses in PEX13-variants carriers. Legend: VLCFAs (very long chain fatty acids): C22:0 docosanoate, C24:0 tetracosanoate, C26:0 hexacosenoate, NA not available. **Additional Figure 1.** Sanger sequencing representative of the p.Arg294Trp variant. Electropherogram of individual A.II-3 (A) showing p.Arg294Trp variant inherited from his father (B). Electropherogram of individual A.II-3 (C) showing  p.Y192QfsTer.14 variant inherited from his mother (D). **Additional Figure 2.** Electroencephalography (EEG) of individual D.II-3. EEG performed at one day of life (A) showing numerous multifocal sharp waves, especially on the temporal regions bilaterally, in the context of continuous electrical activity. EEG control performed at one month of age (B) in the same individual. A slightly hypovolted theta-delta background activity with a reduction in multifocal sharp-waves are observed. **Additional Figure 3.** Fundoscopy examination of individual E.II-1. Note the cherry-red spot of the macula and the diffuse white dots. **Additional Figure 4.** Modelling. Comparison between molecular dynamics simulation snapshots at 0ns (dark) and 250ns (light) for (A) PEX13-WT:PEX14:PEX5 tetramer; (B) PEX13 Arg294Trp:PEX14:PEX5 tetramer. Configurations were obtained by PEX13 homology modelling followed by 500ns of molecular dynamics simulations followed by blind docking to PEX14:PEX5.  Color code: PEX13-WT (green), PEX13-Arg294Trp (magenta), PEX14 (white), PEX5 (blue). Arg and Trp 294 are highlighted in PEX13-WT and PEX13-Arg294Trp, respectively. **Additional Figure 5.** Modelling. A collection of possible homodimeric conformations for (A) PEX13-WT and (B) PEX13-Arg294Trp. Representative conformations of the first eight clusters identified by blind docking. Arg294 is highlighted in PEX13-WT (green) and Trp294 is highlighted in PEX13-Arg294Trp (magenta). **Additional Figure 6.** Closest contact distance distribution. The closest distance between Arg294 (green) [Trp294 (mauve)] and its binding partner in PEX13:PEX13 homodimers calculated over all the generated docking poses.

**Additional file 2: Additional Video 1.** Neurological examination of individual A.II-3. The video showing spastic tetraparesis predominantly affecting the lower limbs and to a lesser extent the upper limbs, dysmetria at finger-to-finger and finger-to-nose tests, Babinski sign and sustained ankle clonus.

**Additional file 3: Additional Video 2.** Deambulation of Individual A.II-3 at 9 years of age. The video showing a few unbalanced steps of individual A.II-3 using a walker. Note the presence of intention tremors and the marked legs intrarotation.

## Data Availability

All data generated or analysed during this study are included in this published article [and its Additional information files]. Further inquiries can be directed to the corresponding author/s.
