# Supplementary material for: Genotype–phenotype correlations and disease mechanisms in PEX13-related Zellweger spectrum disorders
Source: Orphanet J Rare Dis. 2022 Jul 19;17:286. doi: 10.1186/s13023-022-02415-5 (PMC9295491; doi:10.1186/s13023-022-02415-5)
Supplement: Supplementary file 1 — Additional file 1: Additional Table 1. Plasma biochemical analyses in PEX13-variants carriers. Legend: VLCFAs (very long chain fatty acids): C22:0 docosanoate, C24:0 tetracosanoate, C26:0 hexacosenoate, NA not available. Additional Figure 1. Sanger sequencing representative of the p.Arg294Trp variant. Electropherogram of individual A.II-3 (A) showing p.Arg294Trp variant inherited from his father (B). Electropherogram of individual A.II-3 (C) showing p.Y192QfsTer.14 variant inherited from his mother (D). Additional Figure 2. Electroencephalography (EEG) of individual D.II-3. EEG performed at one day of life (A) showing numerous multifocal sharp waves, especially on the temporal regions bilaterally, in the context of continuous electrical activity. EEG control performed at one month of age (B) in the same individual. A slightly hypovolted theta-delta background activity with a reduction in multifocal sharp-waves are observed. Additional Figure 3. Fundoscopy examination of individual E.II-1. Note the cherry-red spot of the macula and the diffuse white dots. Additional Figure 4. Modelling. Comparison between molecular dynamics simulation snapshots at 0ns (dark) and 250ns (light) for (A) PEX13-WT:PEX14:PEX5 tetramer; (B) PEX13 Arg294Trp:PEX14:PEX5 tetramer. Configurations were obtained by PEX13 homology modelling followed by 500ns of molecular dynamics simulations followed by blind docking to PEX14:PEX5. Color code: PEX13-WT (green), PEX13-Arg294Trp (magenta), PEX14 (white), PEX5 (blue). Arg and Trp 294 are highlighted in PEX13-WT and PEX13-Arg294Trp, respectively. Additional Figure 5. Modelling. A collection of possible homodimeric conformations for (A) PEX13-WT and (B) PEX13-Arg294Trp. Representative conformations of the first eight clusters identified by blind docking. Arg294 is highlighted in PEX13-WT (green) and Trp294 is highlighted in PEX13-Arg294Trp (magenta). Additional Figure 6. Closest contact distance distribution. The closest distance between Arg294 (green) [file 13023_2022_2415_MOESM1_ESM.docx]

**Additional Files**

| **VLCFAs** | **Individual A.II-3** | **Individual B.II-1** | **Individual C.II-3** | **Individual C.II-2** | **Individual D.II-3** | **Individual E.II-1** |
| --- | --- | --- | --- | --- | --- | --- |
| **Plasma**  **C22:0** (μmol/L)  **C24:0** (μmol/L)  **C26:0** (μmol/L)  **C26:0/C22:0, ratio**  **C24:0/C22:0, ratio**  **Pristanic acid** (μmol/L)  **Phytanic acid** (μmol/L) | 53.3  58.1  1.01  0.01  1.09  0.04  0.29 | NA  NA  NA  NA  NA  NA  NA | 32.95  28.85  0.24  0.007  0.88  0.10  0.17 | 45.67  39.62  0.43  0.009  0.87  0.10  0.41 | NA  NA  NA  NA  NA  NA  NA | NA  NA  NA  NA  NA  NA  NA |

**Additional Table 1. Plasma biochemical analyses in *PEX13*-variants carriers**

Legend: *VLCFAs* (very long chain fatty acids): *C22:0* docosanoate, C24:0 tetracosanoate, *C26:0* hexacosenoate, *NA* not available.

**Additional Figure 1. Sanger sequencing** **representative of the p.Arg294Trp variant.** Electropherogram of individual A.II-3 (A) showing p.Arg294Trp variant inherited from his father (B). Electropherogram of individual A.II-3 (C) showing p.Y192QfsTer.14 variant inherited from his mother (D).

**
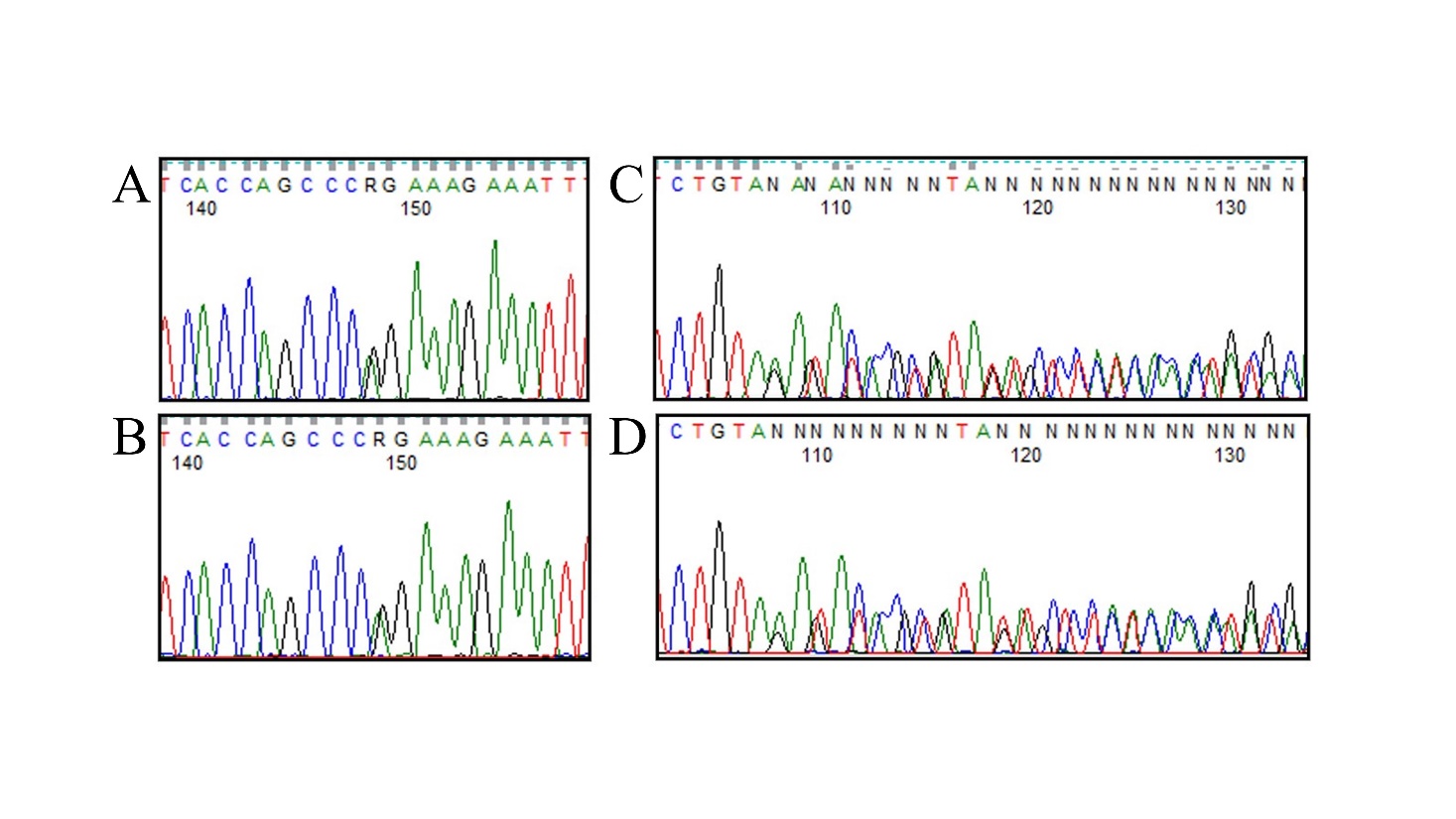
**

**
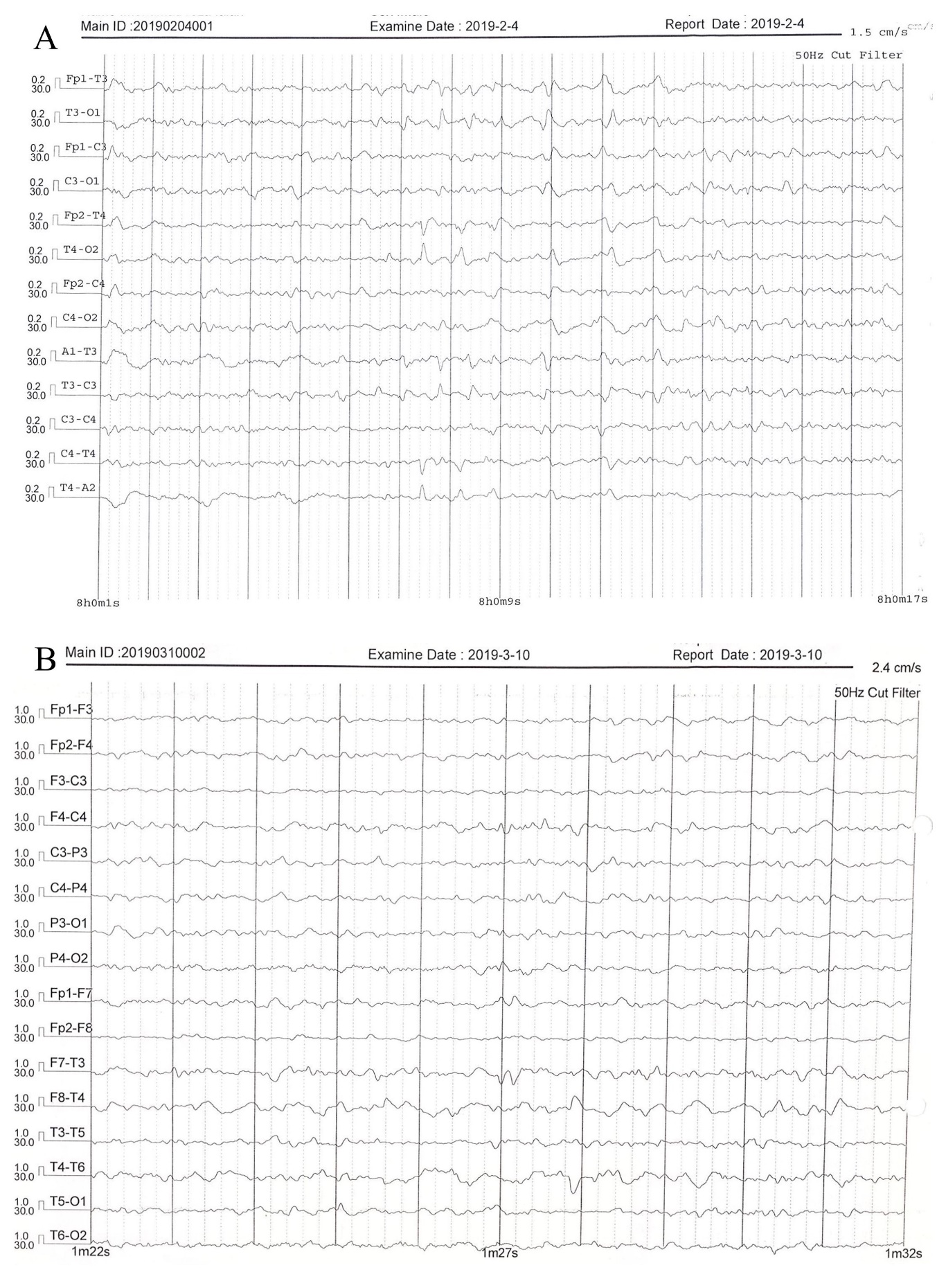
Additional Figure 2. Electroencephalography (EEG) of individual D.II-3.** EEG performed at one day of life (A) showing numerous multifocal sharp waves, especially on the temporal regions bilaterally, in the context of continuous electrical activity. EEG control performed at one month of age (B) in the same individual. A slightly hypovolted theta-delta background activity with a reduction in multifocal sharp-waves are observed.

**Additional Figure 3. Fundoscopy examination of individual E.II-1.** Note the cherry-red spot of the macula and the diffuse white dots.


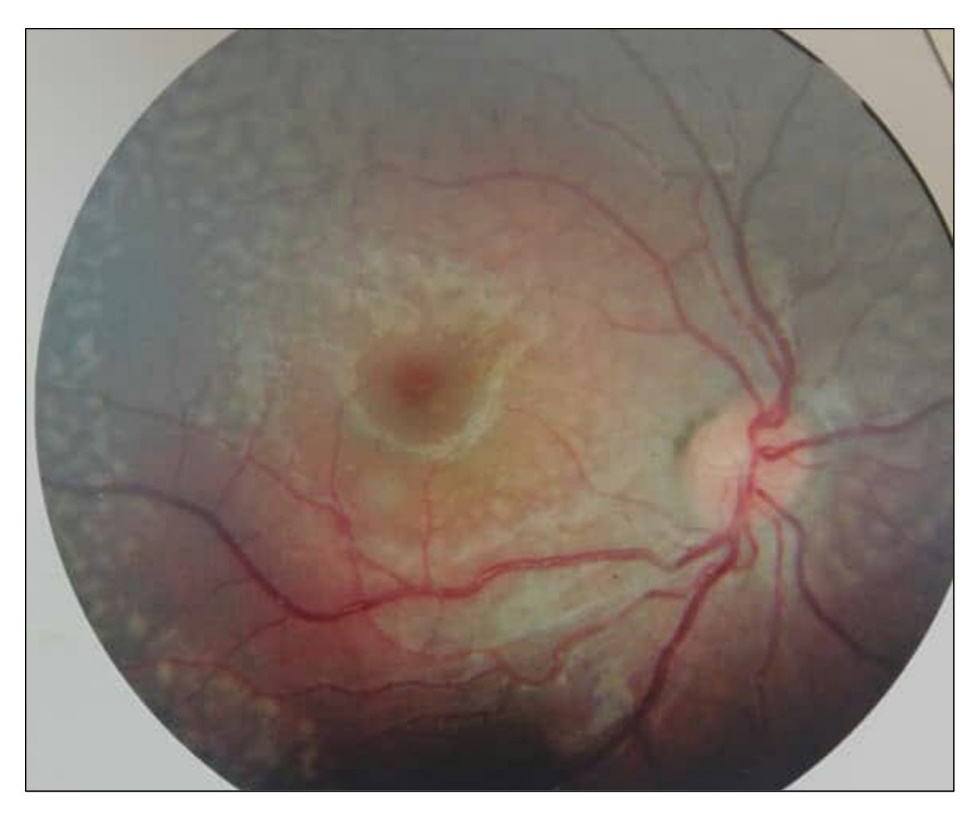


**Additional Figure 4. Modelling.** Comparison between molecular dynamics simulation snapshots at 0ns (dark) and 250ns (light) for (A) PEX13-WT:PEX14:PEX5 tetramer; (B) PEX13 Arg294Trp:PEX14:PEX5 tetramer. Configurations were obtained by PEX13 homology modelling followed by 500ns of molecular dynamics simulations followed by blind docking to PEX14:PEX5. Color code: PEX13-WT (green), PEX13-Arg294Trp (magenta), PEX14 (white), PEX5 (blue). Arg and Trp 294 are highlighted in PEX13-WT and PEX13-Arg294Trp, respectively.


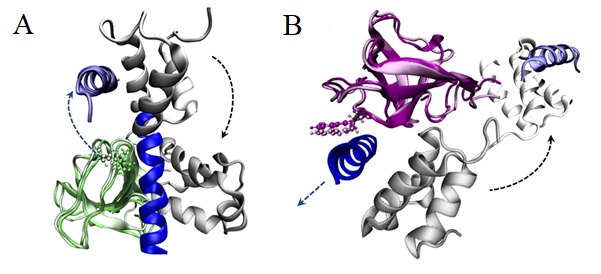


**Additional Figure 5. Modelling.** A collection of possible homodimeric conformations for (A) PEX13-WT and (B) PEX13-Arg294Trp. Representative conformations of the first eight clusters identified by blind docking. Arg294 is highlighted in PEX13-WT (green) and Trp294 is highlighted in PEX13-Arg294Trp (magenta).

**
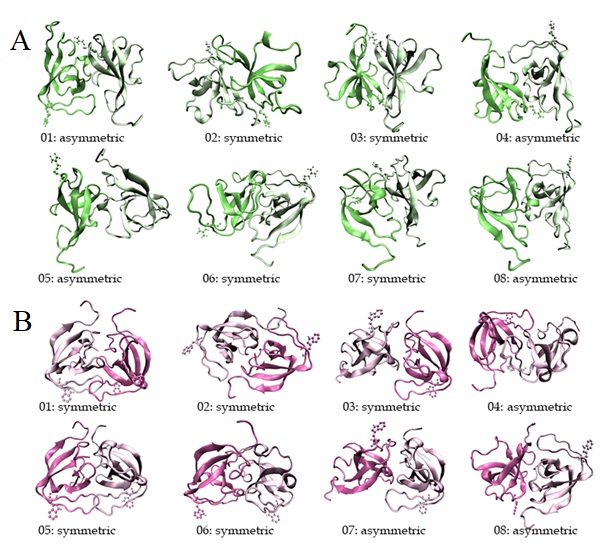
**

**Additional Figure 6. Closest contact distance distribution.** The closest distance between Arg294 (green) [Trp294 (mauve)] and its binding partner in PEX13:PEX13 homodimers calculated over all the generated docking poses.


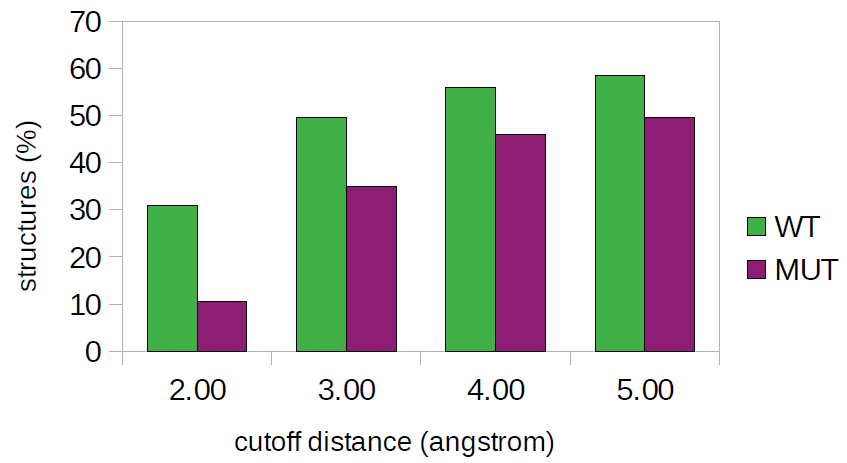


**Additional Video 1. Neurological examination of individual A.II-3.** The video showing spastic tetraparesis predominantly affecting the lower limbs and to a lesser extent the upper limbs, dysmetria at finger-to-finger and finger-to-nose tests, Babinski sign and sustained ankle clonus.

**Additional Video 2. Deambulation of Individual A.II-3 at 9 years of age.** The video showing a few unbalanced steps of individual A.II-3 using a walker. Note the presence of intention tremors and the marked legs intrarotation.
